# Supplementary material for: The interactome of intact mitochondria by cross-linking mass spectrometry provides evidence for coexisting respiratory supercomplexes
Source: Mol Cell Proteomics. 2017 Dec 8;17(2):216–32. doi: 10.1074/mcp.RA117.000470 (PMC5795388; doi:10.1074/mcp.RA117.000470)
Supplement: Supplemental Data [file supp_RA117.000470_133922_0_supp_23978_fzffwf.zip › spectra_annotation/mito_spectra_annotation/366-1-6-1-3-1.pdf]

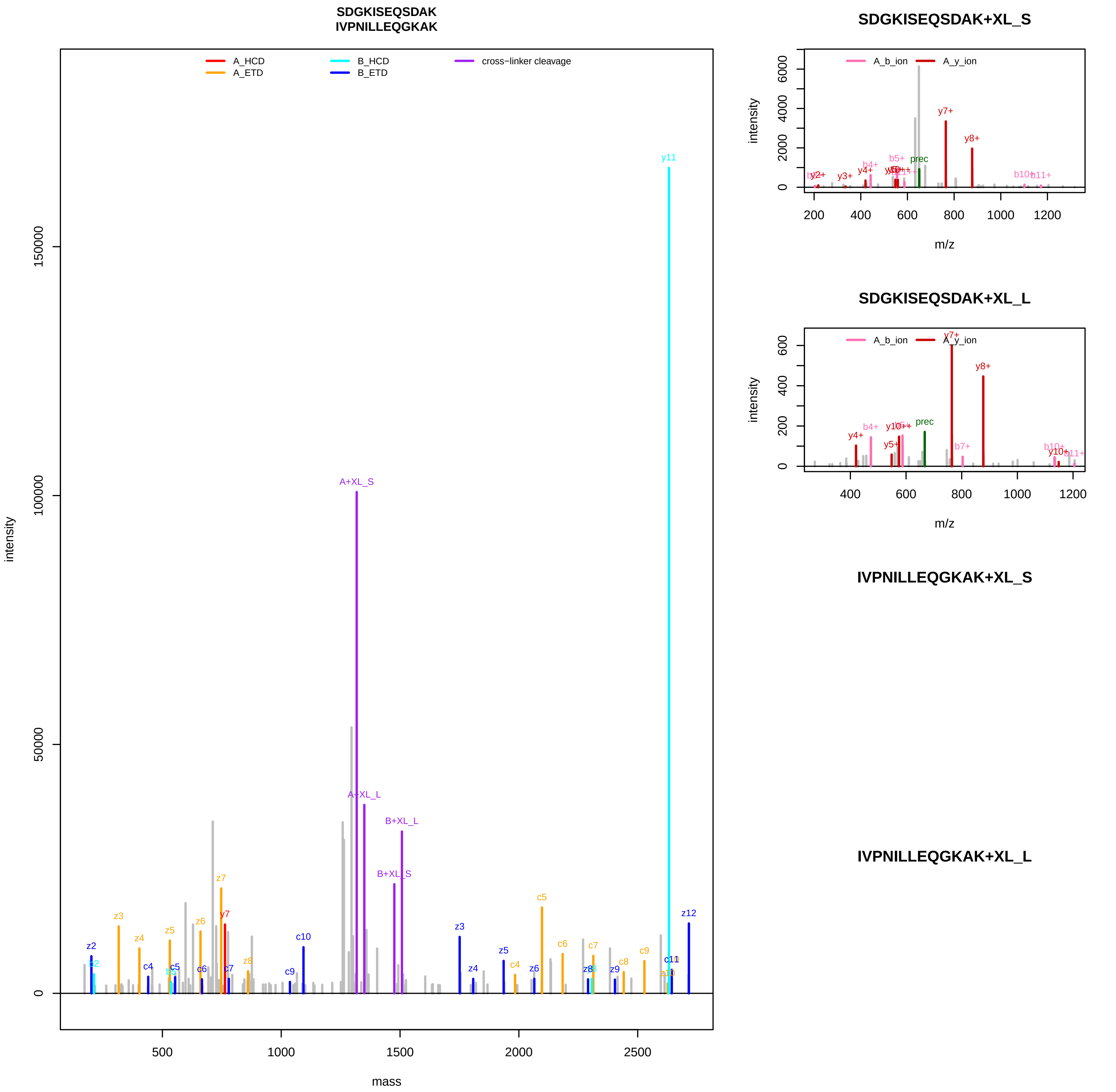
SDGKISEQSDAK
IVPNILLEQGKAK
A_HCD
A_ETD
B_HCD
B_ETD
cross−linker cleavage
intensity
mass
0
50000
100000
150000
500
1000
1500
2000
2500
y11
A+XL_S
A+XL_L
B+XL_L
B+XL_S
z2
c2
z3
z4
c4
b5
z5
c5
c6
z6
z7
y7
c7
z8
c9
c10
z3
z4
z5
c4
z6
c5
c6
c7
z8
c8
z9
c9
c11
z10
z12
SDGKISEQSDAK+XL_S
A_b_ion
A_y_ion
intensity
m/z
0
2000
4000
6000
200
400
600
800
1000
1200
y2+
y3+
y4+
b4+
b5+
prec
y7+
y8+
b10+
b11+
SDGKISEQSDAK+XL_L
A_b_ion
A_y_ion
intensity
m/z
0
200
400
600
400
600
800
1000
1200
y7+
y8+
prec
y4+
b4+
y10++
y5+
b7+
b10+
y10+
b11+
IVPNILLEQGKAK+XL_S
IVPNILLEQGKAK+XL_L
